# Photosynthetic Constraints on Fuel from Microbes

**DOI:** 10.3389/fbioe.2015.00036

**Published:** 2015-03-18

**Authors:** Charles A. R. Cotton, Jeffrey S. Douglass, Sven De Causmaecker, Katharina Brinkert, Tanai Cardona, Andrea Fantuzzi, A. William Rutherford, James W. Murray

**Affiliations:** ^1^Department of Life Sciences, Imperial College London, London, UK

**Keywords:** photosynthesis, photosynthetic efficiency, biofuel, algal biofuel, EROI, light harvesting, rubisco, chlorophyll

## The World Energy Problem and the Biofuel Energy Problem

Oxygenic photosynthesis has been promoted as a system for fuel production on a global scale to replace fossil fuels. The fundamental requirement for this to be viable is that the energy output of the system must be greater than the energy input from fossil fuels. For biofuel production, this criterion is not always met. This issue is often dodged because life-cycle analyses are complex (and thus disputed) and future technological innovations can always be invoked. The second requirement is a sufficiently high rate of solar energy conversion to make the process feasible in terms of the time and space needed to produce fuel on a relevant scale. Both requirements are closely linked to the photosynthetic efficiency; i.e., the conversion efficiency of solar energy to organic material (sugar, biomass, hydrocarbons, etc.). Here, we discuss limitations on photosynthetic efficiency and approaches suggested to overcome them. We focus on biofuels produced by photosynthetic microbes as they are often considered the fuels of the future for their year-round cultivation, non-competition with food crops, higher reported photosynthetic yields, and the potential for genetic engineering to produce fuels directly (Brennan and Owende, 2010).

The energy investment required for biomass production (e.g., water, nutrients, fertilizers, stirring, bubbling, containment, harvesting, processing) cancels out some or all of the energy gained from sunlight (Slade and Bauen, 2013). This is described by the energy returned as a proportion of energy invested (EROI), and this factor is the key measure of energy sustainability in life-cycle analysis (Murphy and Hall, 2010). If the EROI is >1, the system produces a fuel with net solar energy content; if <1 it does not: it wastes fossil fuel (Hall et al., 2014). The energy investment in bioreactors, required to achieve the highest photosynthetic conversion efficiencies (Cuaresma et al., 2009), cancels out all of the gain in energy returned and reduces the EROI below 1 (Resurreccion et al., 2012). While EROI values are better for low-input open pond systems, they still lie between 0.4 and −1.1 in multiple life-cycle analyses (Zaimes and Khanna, 2013; also Resurreccion et al., 2012) [c.f. ~20 for oil and ~84 for hydroelectric (Hall et al., 2014)]. Furthermore, the energy required for processing biomass into liquid biofuel decreases the EROI.

Instead of producing biomass, photosynthetic microbes that continuously excrete biofuel or biofuel precursors have been genetically engineered (Nozzi et al., 2013). If the product can be easily isolated, and if the microbe diverts energy captured from the sun away from growth and into the product, this would have advantages over conventional approaches. Given the relative novelty and commercial interest in such approaches, independent life-cycle analyses are not yet available (Moheimani and McHenry, 2013); however, the intrinsic limitations of photosynthesis will still apply. The production of volatile fuels, the use of genetically modified strains, and the prevention of contamination would all seem to call for the use of closed photobioreactors, thus incurring a serious energy cost (Resurreccion et al., 2012; Slade and Bauen, 2013).

Scaling-up of the process in order to produce enough fuel to replace fossil fuels is a major problem, as energetic and material costs are not always linear. Serious challenges for scalability include water usage, nitrogen and phosphorus recovery, and a range of environmental and ecological issues (Clarens et al., 2010; Chisti, 2013; Benson et al., 2014). However, meeting both energy prerequisites (an EROI significantly >1 and a high rate of solar energy conversion) on a pilot scale seems advisable before scaling-up is considered.

The energetic prerequisites rely fundamentally on the efficiency of photosynthesis. Calculations for theoretical photosynthetic efficiency agree on a maximum value for solar energy to carbon–carbon bonds in glucose of around 13%, falling to around 5% of solar energy to biomass for C3 plants, considering photorespiratory and respiratory losses (Zhu et al., 2010). The highest efficiency reported for photosynthetic microbes under controlled lab conditions is 3% for light-to-biomass [Melis, 2009; also Cuaresma et al. (2009)]. Under growth conditions more relevant to industrial settings, the efficiency is stated to be significantly lower than this (Melis, 2009). Efforts are thus being made to find ways of improving photosynthesis itself.

## Engineering to Improve Light Collection

The first stage of photosynthesis is light-capture by pigments in the antenna complexes which deliver excitation energy to the chlorophylls of photochemical reaction centers (Croce and van Amerongen, 2014). Antenna pigments greatly outnumber the reaction center chlorophylls so that the reaction centers can obtain excitations from light over a much bigger surface area. Complex regulatory mechanisms, which convert excess energy into heat when reaction centers become saturated, are activated at surprisingly low photon fluxes, resulting in the majority of absorbed photons being wasted.

In principle, reducing the antenna size should alleviate this problem by reducing saturation and the related shading problem (Nakajima and Ueda, 1997). In practice, some antennae mutants do show moderate improvement in photosynthetic growth [e.g., Mussgnug et al. (2007) and Kirst et al. (2014)]. This has only been demonstrated under a narrow range of growth conditions (high light, high cell density, low CO_2_ concentration), and lower productivity is reported under other conditions [e.g., Page et al. (2012), Kirst et al. (2014), and Lea-Smith et al. (2014)].

Antennae truncation can improve photosynthetic yields under specific, controlled conditions. The improvement gained should be considered as moving, slightly, toward the 13% limit rather than extending that limit. The requirement for special growth conditions likely cancels these gains.

## Engineering to Improve Photochemistry

Photosynthetic pigments absorb from 400 to 700 nm, less than half of the solar spectrum. Longer-wavelength pigments could be used to extend this range (Blankenship et al., 2011). Extending absorption out to 750 nm would result in an increase in the number of available photons by 19% (Chen and Blankenship, 2011) and, in principle, could allow a small increase in the theoretical 13% limit.

Organisms containing longer-wavelength chlorophylls *d* and *f* naturally occur (Chen et al., 2010; Gan et al., 2014). While chlorophyll *d* is involved in primary charge separation in both photosystem I and photosystem II (PSII) in *Acaryochloris marina* (Renger and Schlodder, 2008), it is not yet known if chlorophyll *f* plays a role in reaction center photochemistry. The energy of the 710 nm photon absorbed by chlorophyll *d* is 70 meV, less than that in the 680 nm photon (1.82 eV) absorbed by chlorophyll *a*. Given the stringent energy requirements for water oxidation, this is expected to result in an increase in the probability of back-reactions leading to singlet oxygen-induced damage (Rutherford et al., 2012). While this does not impact on the efficiency of growth under optimized conditions (Mielke et al., 2011), it may make PSII more susceptible to photodamage under variable light conditions. A 750 nm pigment in PSII would decrease the energy available by 170 meV with an even greater risk of back-reaction-mediated oxidative damage and associated metabolic costs. When photosynthesis using far-red pigments occurs in nature, it does so in restricted environments (Chen et al., 2010; Gan et al., 2014). In applications aimed at fuel production, more of the spectrum may be accessed but it would likely require tightly controlled growth conditions and thus cost energy.

## Engineering to Improve Carbon Fixation

Ribulose 1,5-bisphosphate carboxylase/oxygenase (Rubisco) is the enzyme responsible for carbon fixation in the Calvin cycle. This enzyme is slow and inefficient (Savir et al., 2010). While there is a variety of different Rubiscos in nature (Tabita et al., 2008), there is a suggested trade-off between CO_2_ affinity and carboxylation velocity, limited by structural or biochemical characteristics of the enzyme (Tcherkez et al., 2006; Savir et al., 2010). Recently, directed evolution of a cyanobacterial Rubisco produced a mutant with 2.9-fold improvement in activity with only a 9% loss in CO_2_/O_2_ specificity. *Synechocystis* sp. PCC 6803 (henceforth *Synechocystis*) expressing the improved Rubisco did not show growth improvements, instead producing 25% less Rubisco (Durão et al., 2015). This is consistent with the finding that altering Rubisco activity has little effect on the rate of photosynthesis in *Synechocystis* (Marcus et al., 2011). Results under conditions of high CO_2_ are different (Atsumi et al., 2009).

Photorespiration is the set of biochemical pathways which starts with Rubisco oxygenation activity and recycles the product 2-phosphoglycolate. Photorespiration is considered an energetic cost. While reducing photorespiratory losses can yield large growth improvement in plants (Kebeish et al., 2007), current efforts to improve recovery of photorespiratory metabolites in cyanobacteria show no change in growth (Shih et al., 2014). This may be due to carbon-concentrating mechanisms in cyanobacteria which protect against high levels of O_2_ around Rubisco, reducing photorespiration (Dou et al., 2008; Mangan and Brenner, 2014). Furthermore, the by-products of photorespiration are managed by three different pathways in *Synechocystis* (c.f. one in plants) (Eisenhut et al., 2008; Peterhansel and Maurino, 2011; Young et al., 2011).

Avoiding Rubisco using alternative aerobic carbon fixation cycles, such as the 3-hydroxypropionate bi-cycle (Zarzycki et al., 2009), or even newly designed pathways (Bar-Even et al., 2010; Erb, 2011), is being attempted to improve carbon fixation.

There is clearly scope for improvement in carbon fixation. Most approaches work within the 13% efficiency limit; however, alternative carbon fixation pathways may require less ATP, potentially extending this limit. Unlike in plants, it is hard to imagine a more effective carbon-concentrating and photorespiratory metabolism than that already found in cyanobacteria.

## Engineering to Influence Downstream Metabolic Pathways

Incorporating biofuel producing pathways into microbial photosynthesizers can be relatively straightforward. Alkanes are made naturally (Han et al., 1968) and strains producing detectable levels of ethanol (Dexter and Fu, 2009), acetone (Zhou et al., 2012), ethylene (Guerrero et al., 2012), isoprene (Lindberg et al., 2010) and 2,3-butanediol (Oliver et al., 2013) among others have been generated (Rosgaard et al., 2012; Nozzi et al., 2013).

In contrast, metabolic engineering to increase product yield is difficult, product-specific, and hard to rationalize when models of central metabolism are still being adjusted (Knoop et al., 2013). It is clear that some progress can be made; for example, isoprene producing strains have recently been improved 2.5-fold by co-expression of the mevalonic acid pathway (Bentley et al., 2014). Even so, standard metabolic engineering approaches such as removing storage pathways require careful attention in photosynthetic organisms (Van der Woude et al., 2014).

## Engineering so it Works: A Holistic Approach to a Complex System

Most efforts directed at improving photosynthetic efficiency focus on the individual components described in previous sections. In contrast, perhaps the most striking improvement on “natural” photosynthesis came from the optimization of source (light energy) versus sink (metabolic capacity). This takes into account the fact that the kinetics of both the light and dark reactions are inherently linked (Paul and Foyer, 2001).

The advantage of source/sink optimization was illustrated by the heterologous expression of the proton/sucrose symporter CscB in *Synechococcus elongatus*. Up to 36.1 mg l^−1^ h^−1^ of sucrose was exported, a record for direct photobiological production (Ducat et al., 2012). These strains also showed increases in PSII activity, carbon fixation, and chlorophyll content under optimized conditions. The export of the photosynthetic sink (sucrose) is critical, suppressing photodamage by helping to maintain an oxidized electron transport chain. In addition, the sucrose biosynthetic pathway is endogenous to *S. elongatus* and does not place heavy demands on either ATP or NADPH.

The high-energy photoexcited states and highly reactive redox intermediates of the light reactions are heavily reliant on regulatory and protective mechanisms to prevent oxidative damage. Mismatches in the source/sink balance could, for example, result in the dark reactions being outrun by the light reactions causing photoinhibition. Specific changes to improve the light or dark reactions independently may be counterproductive if the balance of source to sink is perturbed. Sink-enhanced strains may be the material of choice for future attempts to improve specific aspects of photosynthesis (antenna, pigment optimization, electron transfer efficiency, CO_2_ fixation, and down-stream metabolism).

## Conclusion

**Figure 1:**
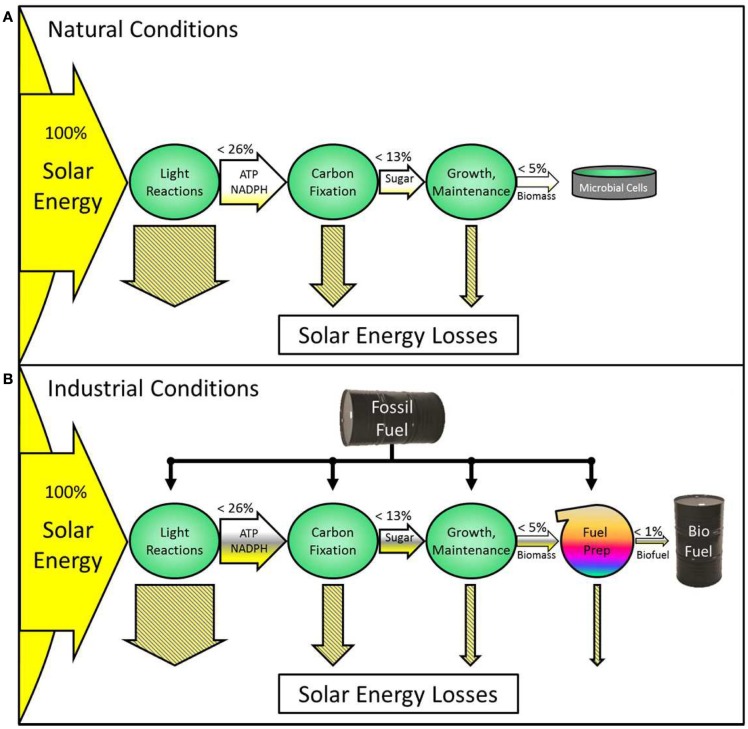
**This diagram illustrates energy flow through a photosynthetic system without fossil fuel energy input (A) and another using fossil fuels (B)**. Horizontal arrows represent the flow of energy through the system, with labels on the arrows showing the type of stored energy at each stage. Theoretical maximum percentages of solar energy retention are written above the arrows for each stage, with yellow fill indicating that achievable levels are lower. Hatched arrows represent solar energy losses. Green circles represent cellular processes. Energy losses in the light reactions include: (1) photons outside the chlorophyll absorbance range, (2) the energy in absorbed photons in excess of that corresponding to the first excited state of the reaction center, and (3) antennae saturation and shading effects; those incurred in the dark reactions include: (1) mismatched substrate stoichiometries (e.g., NADPH, ATP), (2) Rubisco oxygenation reactions, and (3) thermodynamic losses; those in growth include: (1) protein turnover and (2) respiratory losses. **(A)** Biomass grows naturally with no fossil fuel input. The percentage of solar energy converted into the final product is very low (small yellow gradient). **(B)** Fossil fuel is used to control conditions such as temperature, light, CO_2_, sterility, mixing, and other factors. This system produces biofuel. The yield (arrow fill) is higher at each stage, partly due to an increased percentage of solar energy which is retained (yellow fill) and partly due to the energy from fossil fuel (gray fill). Synergistic interactions between engineered strains and controlled environments could increase the fossil-fuel-generated improvements to photosynthetic efficiency and the maximum energy retained. Such systems are only beneficial if the increase in yield is greater than the fossil fuel input. Currently, this condition is rarely met and generally ignored. Using energy from renewable sources such as hydro, photovoltaic, or wind instead of fossil fuels could make a similar conversion system sustainable.

At present, photosynthetic microbial biofuels are not viable in energy terms. This is related to intrinsic inefficiencies in photosynthesis, and thus research has been directed to improving photosynthesis. A brief survey indicates that most suggested modifications would be beneficial only under restricted culture conditions. Controlled growth in bioreactors may then be required but this will incur a significant energy cost, which at present is much bigger than the engineered efficiency gain (see Figure 1). Nevertheless, attempts to improve photosynthesis have not been wholly negative and it is suggested that “sink-maximized” strains should be used for engineering of targeted improvements in order to avoid metabolic congestion and mismatches at the interface between the dark and light reactions.

Given the energetic problems with “algal” biofuels, it may be better to use the technology to produce complex, high-value chemicals (including specialized aviation fuels or their precursors) rather than low-value products such as biomass and ethanol. This, however, becomes a manufacturing process rather than a means of capturing solar energy. The production of fuels made in this way might be economically or strategically viable, but with fossil fuels as the main energy input, this would clearly not be relevant to fossil fuel replacement. These processes could become a sustainable energy conversion and storage technology if alternative energy sources such as hydroelectric, wind, solar, geothermal were used to drive them.

## Conflict of Interest Statement

The authors declare that this research was conducted in the absence of any commercial or financial relationships that could be construed as a potential conflict of interest.
